# Case report: Enfortumab vedotin induced refractory DKA and multi organ failure – a rare fatal adverse event

**DOI:** 10.3389/fonc.2024.1332478

**Published:** 2024-02-15

**Authors:** Astha Koolwal Kapoor, Carleton S. Ellis, Deepali Pandey, Derek B. Allison, Zin W. Myint

**Affiliations:** ^1^ Department of Internal Medicine, Vassar Brothers Medical Center Nuvance Health, Poughkeepsie, NY, United States; ^2^ Markey Cancer Center, University of Kentucky, Lexington, KY, United States; ^3^ Department of Pharmacy, University of Kentucky, Lexington, KY, United States; ^4^ Department of Urology, University of Kentucky, Lexington, KY, United States; ^5^ Department of Pathology and Laboratory Medicine, University of Kentucky, Lexington, KY, United States; ^6^ Department of Internal Medicine, Division of Medical Oncology, University of Kentucky, Lexington, KY, United States

**Keywords:** diabetes ketoacidosis, CRRT, insulin, enfortumab vedotin, maculopapular rash, liver failure

## Abstract

There are very few therapeutic options to treat patients with locally advanced or metastatic Urothelial Cancer (UC). Enfortumab vedotin (EV) was recently approved by the FDA and has become a new therapeutic option for patients previously managed with conventional treatments. Despite its efficacy, EV carries the potential for infrequent yet severe adverse effects. In this report, we present a case of a patient undergoing EV treatment for urothelial carcinoma who developed refractory diabetic ketoacidosis (DKA) unresponsive to escalating insulin doses and necessitating continuous renal replacement therapy. While DKA was resolved, the patient eventually succumbed to progressive maculopapular skin rash, liver failure, and respiratory failure. Additionally, the study delves into a review of cases of EV-induced refractory DKA in the literature, shedding light on the similarities in patient profiles, timelines of adverse effects and the treatment strategies employed to manage the ensuing complications.

## Introduction

Enfortumab vedotin (EV) is an antibody-drug conjugate directed against nectin-4, a protein which is highly expressed on the surface of bladder cancer cells. When EV binds to nectin-4, it is internalized and releases monomethyl auristatin E (MMAE), a potent anti-tumor agent that disrupts microtubules within the cells. This disruption ultimately induces cell apoptosis and leads to cell death (1, 2). The U.S. Food and Drug Administration (FDA) granted accelerated approval to EV in December 2019 and full approval in July 2021 for (i) patients with locally advanced or metastatic urothelial cancer who have progressed on immune check point inhibitors and a platinum-based chemotherapy or (ii) patients who are ineligible for cisplatin-based chemotherapy and have previously received one or more lines of therapy. The latter is based on EV-301, which was a global, phase 3 randomized trial comparing EV to investigator’s choice chemotherapy for patients with locally advanced or metastatic urothelial carcinoma. This study demonstrated significant improvements in both progression free survival and overall survival with EV (3–5).

Herein, we describe a patient with metastatic urothelial carcinoma being treated with EV who presented with severe, refractory diabetic ketoacidosis (DKA) requiring admission to the intensive care unit (ICU) for continuous insulin infusion and continual renal replacement therapy (CRRT). The patient’s DKA ultimately resolved, however, later in the admission, he developed a maculopapular skin rash, fever, liver injury, and respiratory failure, which led to a fatal outcome.

## Patient case

**Figure 1:**
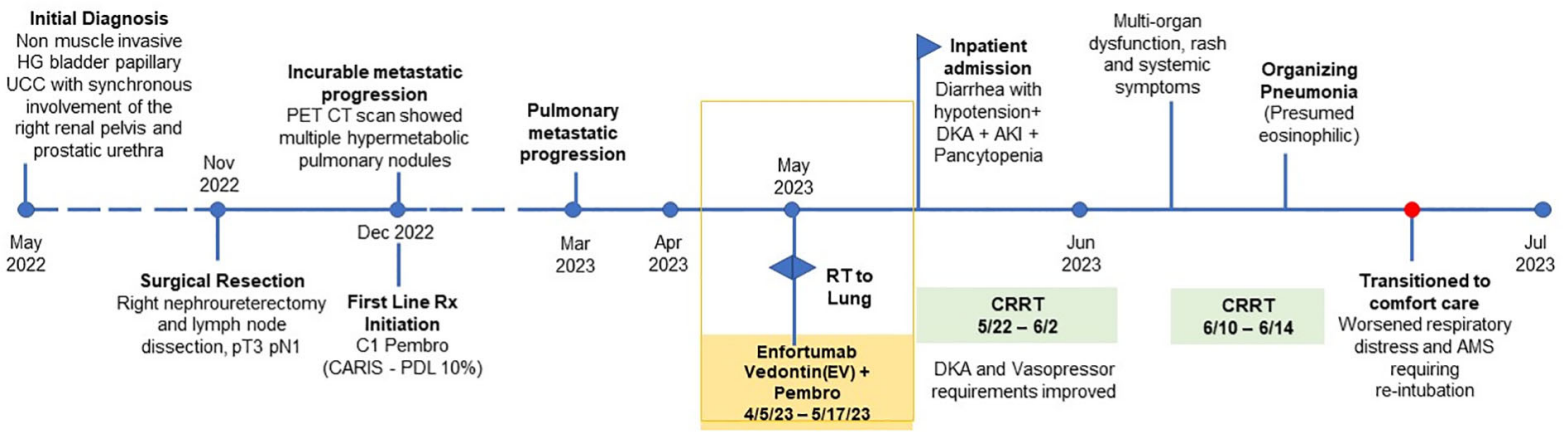
Timeline of the patient’s cancer treatment.

Our patient is a 71-year-old gentleman with a past medical history of prediabetes (A1c 6.1% requiring no anti-diabetic medications), hypertension, hyperlipidemia, stage 3 chronic kidney disease (CKD), a remote history of coronary artery disease (CAD), and recurrent urothelial carcinoma metastatic to bilateral lungs. He progressed on first-line platinum-based chemotherapy and quickly progressed through second-line immunotherapy (pembrolizumab). Next generation sequencing showed no actionable mutations while PD-L1 (22c3) immunohistochemistry was positive with a Combined Positive Score (CPS) of 10. Due to a lack of other options, he was subsequently started on EV while continuing immunotherapy. His oncology treatment timeline is shown in **Figure 1**. Five days after his third infusion of EV, he presented to the emergency department with generalized weakness, diarrhea (up to 10 episodes per day), shortness of breath on exertion and at rest, fatigue, lethargy, and decreased food intake. He was found to be in hypovolemic shock, was admitted to the ICU, and was started on vasopressors. His cortisol level was 16.6 mcg/dL (reference: 5 – 25 mcg/dL). Thus, adrenal insufficiency was ruled out.

**Figure 2:**
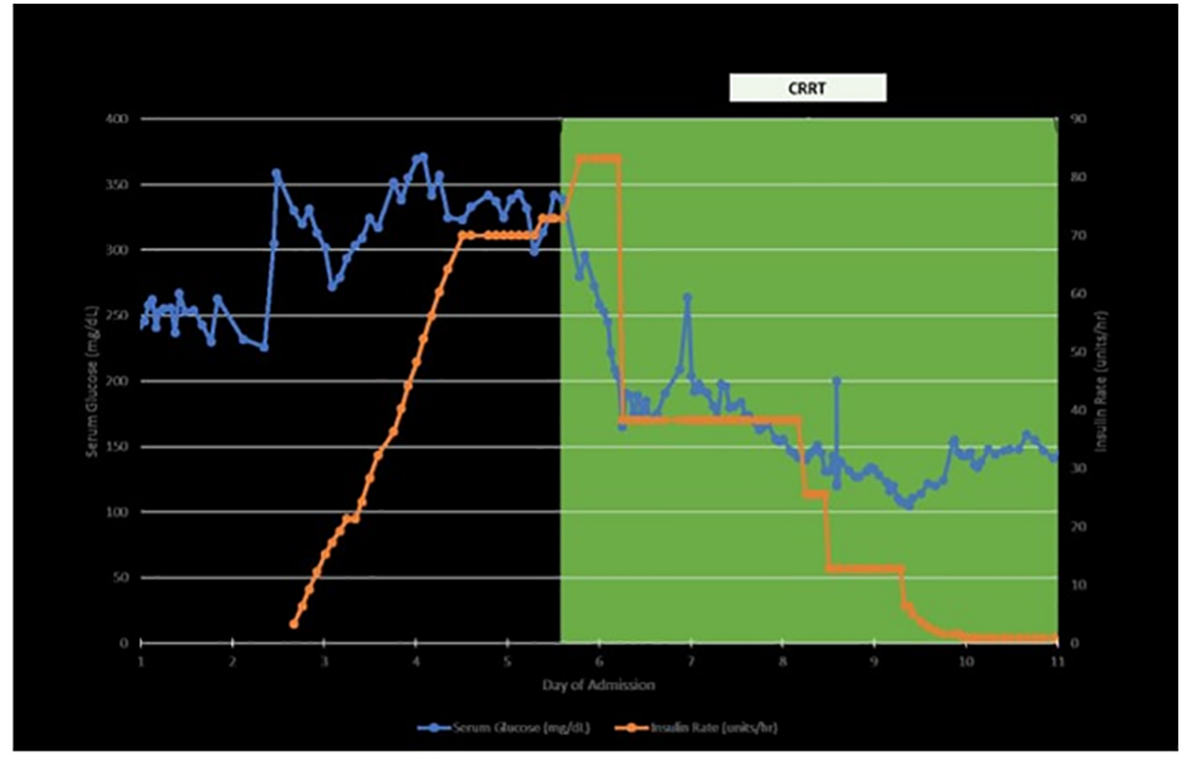
Timelines of insulin requirement, blood sugar level and CRRT (continuous renal replacement therapy).

Other pertinent labs revealed Na 128 (reference: 135 – 145 mEq/L), CO2 14 (reference: 23- 29 mEq/L), Cr 3.44 (baseline 1.5), pH 7.29 (reference: 7.35 – 7.45), uric acid 11.3 (reference: 3.7 – 8.0 mg/dL), beta-hydroxybutyric acid 1.76 (reference: ≤0.27 mmol/L), serum ketones and urine ketones positive (reference: normal), LDH 258 (reference: 116 – 250), A1c 6.1%, anion gap 16 (reference: 6 – 12 mEq/L), glucose 331 mg/dL (reference: 70 – 100), C-peptide >30 (reference: 0.9 – 1.8 ng/ml), anti-glutamic acid decarboxylase (anti-GAD) 3 U/ml (reference: 0-4.9 U/ml) and normal LFTs. Comprehensive GI panel was negative. CT abdomen and pelvis was unremarkable. Diarrhea resolved spontaneously without the administration of steroids. His diarrhea was most likely attributed to enfortumab vedotin. Based on the above lab findings, he was started on a continuous insulin infusion drip for diabetes ketoacidosis (DKA). He required a maximum rate of insulin drip 90 units/hour continuously for a total of 9 days until his glucose level reached <150 mg/dL and was then transitioned to regular insulin (**Figure 2**). On Day 4, continuous renal replacement therapy (CRRT) was initiated. His glucose level was significantly improved after CRRT was initiated and it was continued for a total of 15 days (**Figure 2**). On Day 15, CRRT was discontinued and he was able to be weaned off of vasopressors. On Day 17, his bicarbonate was normalized and he was downgraded from the ICU.

**Figure 3:**
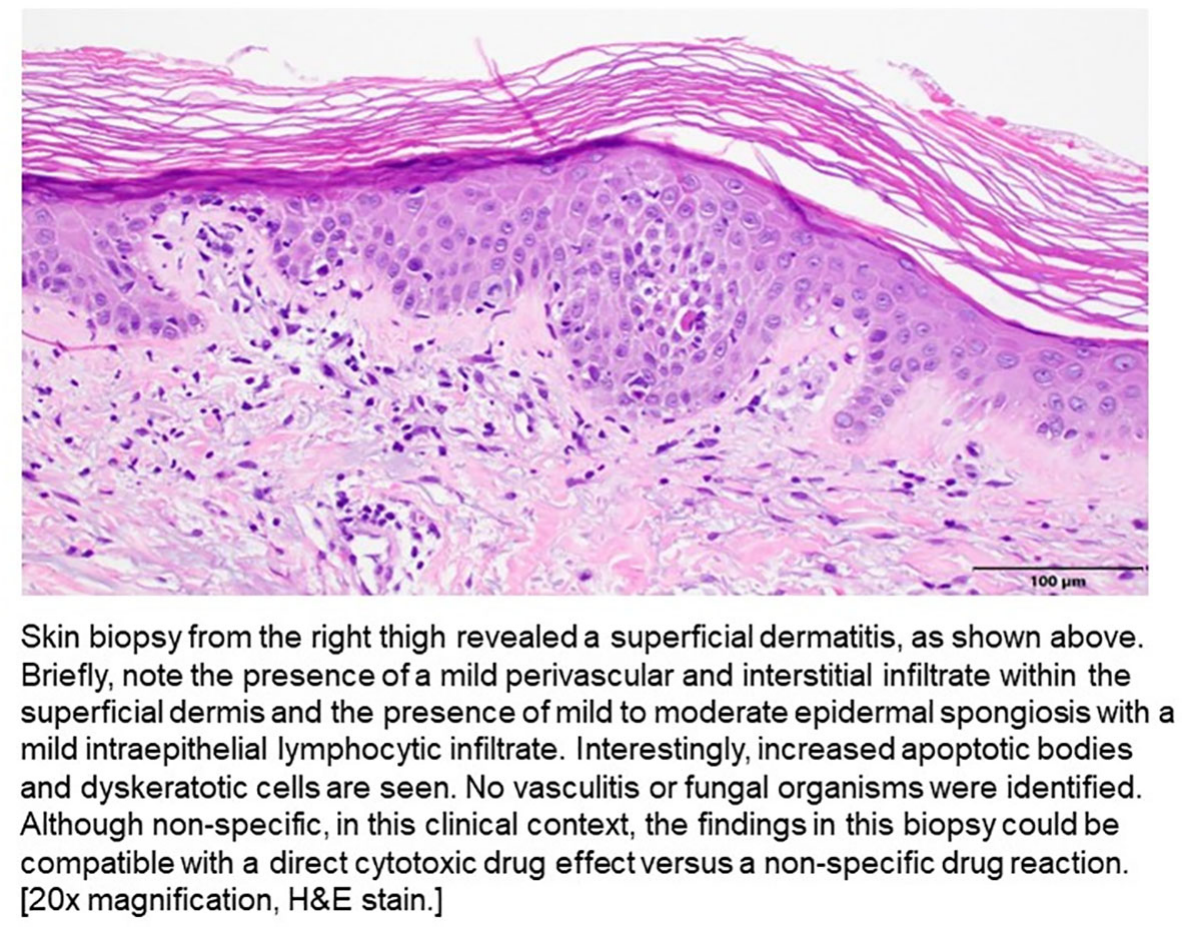
Histopathologic findings on skin biopsy.

On Day 19, his LFTs began to rise, and he developed an intermittent fever (highest temp >102 F or 38.8°C), jaundice, a diffuse maculopapular rash, hypoxia, and became more lethargic. He was transferred back to the ICU, was re-intubated, and CRRT was re-initiated. He underwent ERCP and no biliary obstruction was identified. A punch biopsy was obtained and pathology demonstrated a superficial dermatitis with increased apoptotic bodies and dyskeratotic cells, which could be compatible with a direct cytotoxic drug effect versus a non-specific drug reaction (**Figure 3**). Vasculitis was not seen. High dose prednisone and broad spectrum antibiotics were initiated given the skin rash, fever, and transaminitis. He did not have eosinophilia nor lymphocytosis.

He was able to be extubated, however, shortly after extubation, he became more hypoxic with concern for pneumonia. Chest imaging showed scattered bilateral ground glass opacities. An infectious workup was negative, including studies for COVID-19, HSV1, HSV2, fungal serology, and bronchoalveolar lavage cytology. He required re-intubation; however, his family did not want to pursue any further aggressive therapy given his complicated hospitalization/impending decline. He passed away peacefully two days later.

## Discussion

Diabetic ketoacidosis (DKA) is one of the potential complications that can develop in patients receiving EV, whether or not they have pre-existing diabetes. While it is acknowledged that the side effects of pembrolizumab and enfortumab vedotin can overlap, the rarity of immunotherapy induced hyperglycemia is highlighted, reported in only 0.1% of patients (6). In contrast, in the phase II EV-201 study, which involved the use of EV after immunotherapy in cisplatin-ineligible patients with advanced urothelial carcinoma, grade 3-4 hyperglycemia was observed in 7% of patients (3). DKA related fatality occurred in less than 0.1% of the patients in that study (3). In a lecture review by Cheema A et al. on pembrolizumab-induced autoimmune diabetes, six case reports were cited. Notably, 50% of these patients had anti-GAD antibodies positive. In our patient, anti-GAD antibody was negative (7).

We performed a PubMed comprehensive literature review using the search terms “Enfortumab vedotin” and “Diabetes Ketoacidosis”, “Hyperglycemia”. We reviewed all results and found the following previous case reports as outlined below (**Table 1**). Most patients presented within one week of administration of either the second or third EV infusion, like our patient. However, it is crucial to acknowledge that the onset of immune-related adverse events can vary significantly ranging from weeks to months after the initial dose of treatment, spanning between cycle 2 and cycle 17 (7).

**Table 1:** Cases of Enfortumab vedotin induced refractory diabetic ketoacidosis.

Reference #	Age	Sex	Diagnosis	Other Complications	Previous Therapies	EV cycles	CRRT	ICU Admission	Insulin dosing required	Outcome
(8)	59	Female	Urothelial cancer	None	NA	Third	No	Unknown	60 units/hour	Discharged
(9)	72	Male	Metastatic urothelial cancer	None	NA	Unknown	Yes	Yes	80 units/hour	Expired
(10)	69	Male	Metastatic urothelial cancer	Shock AKI TEN	NA	Unknown	Yes	Yes	Unknown	Expired
(11)	75	Male	Metastatic urothelial cancer	Undifferentiated shock + MODS	Nivolumab and Ipilimumab	Second	Yes	Yes	Up to 1000 units a day	Expired

*EV, enfortumab vedotin; NA, not available; CRRT, continuous renal replacement therapy; AKI, acute kidney injury; TEN, toxic epidermal necrolysis; MODS, multiple organ dysfunction syndrome.

Furthermore, most cases required ICU admission and required excessive amounts of insulin per day without much influence on the patient’s blood glucose, as we observed. Of note, the blood glucose remained in the 300-400 range despite maximum efforts to control it with large amounts of insulin, generally warranting initiation of CRRT.

In addition, most cases reported serious fatal outcomes and the cause of death was attributed to DKA and renal failure. In our patient, a distinctive presentation involving skin rash, liver injury, and pulmonary failure was also observed. Our working hypothesis revolves around the potential presence of nectin-4 in these organs. Such a presentation of multi-organ involvement is infrequent in immune-mediated adverse events, underscoring the unique and complex nature of this case. Considering the factors mentioned above, we believe that our patient’s DKA and other organs involvement are predominantly associated with enfortumab vedotin rather than pembrolizumab.

Our patient’s DKA resolved after a prolonged ICU admission, and his clinical symptoms were improved two weeks after being treated with insulin and CRRT. This underscores the crucial significance of early recognition of EV-induced DKA and, more notably, the urgency of early intervention with CRRT to improve patient outcomes.

The exact mechanism by which EV induces DKA remains unclear, but some speculations have been made. One theory suggests that EV might interfere with the PI3K/mTOR/AKT pathway, leading to profound insulin resistance in the insulin pathway (8). Additionally, it was noted in the FDA submission for EV that there was a significant increase in glucose uptake in human skeletal muscle cells and decline in insulin secretion from human islet cells following exposure to MMAE, the latter resulting in hyperglycemia (12). In our case, it is assumed that the hyperglycemia was due to insulin resistance because high doses of insulin were required in an insulin-naïve patient. As a result, further research is required to fully understand the precise biological processes involved. In one study, it was observed that the duration of clearance of EV from the system seemed to be associated with the patient’s recovery from insulin therapy (8). This association suggests that discontinuing EV treatment may alleviate the insulin resistance, while the early initiation of CRRT can aid in the removal of glucose and EV, thus leading to an improvement in the patient’s condition.

The complications associated with EV appear to be intricately linked to its mechanism of action and its influence on cells that express high levels of the target molecule, nectin-4 (13, 14). Although nectin-4 overexpression is not exclusive to bladder cancer, the interplay between EV and nectin-4 can yield both therapeutic benefits and unintended side effects. Notably, these complications may be associated with nectin-4 binding in various sites (13, 14). Our patient eventually developed skin rash, liver failure, and respiratory failure. Ultimately, he died from multi-organ failure most likely related to complications secondary to EV.

Skin-related issues such as rashes may arise due to EV’s interactions with nectin-4 in epidermal cells, where nectin-4 is highly expressed (15, 16). The presentation of EV-related dermatologic events is quite variable, including its onset, distribution, morphology, and severity. The most common and well-recognized adverse events of EV is maculopapular rash. More rare but severe dermatologic reactions such as Stevens-Johnson syndrome (SJS) and Toxic Epidermal Necrolysis (TEN) are also known to occur and are included in the boxed warning of the US prescribing information for EV (13).

Furthermore, alternations in liver, as evidenced by laboratory test in our patient, might be influenced by the presence of nectin-4 in these organs, possibly affected by EV. It is noteworthy to consider that the respiratory failure may have been attributed to pneumonia, despite the negative results from the infectious workup, or potentially due to disease progression in his lung metastases. Furthermore, it is worth mentioning that certain respiratory cells could have exhibit nectin-4 expression (16), suggesting a possible link to the observed respiratory distress. Published data indicate that EV-induced pneumonitis has been reported in approximately 25% of the patient based on two prospective trials (17).

These complexities underscore the delicate balance between therapeutic efficacy and unwanted adverse effects in the context of targeted therapies like EV. Further investigation is needed to gain a comprehensive understanding of the underlying mechanisms and optimize the management of patients undergoing EV therapy.

## Data availability statement

The raw data supporting the conclusions of this article will be made available by the authors, without undue reservation.

## Ethics statement

Written informed consent was obtained from the family member for the publication of any potentially identifiable images or data included in this article.

## Author contributions

AK: Writing – original draft, Writing – review & editing, Conceptualization, Data curation. CE: Writing – review & editing. DP: Writing – review & editing. DA: Writing – review & editing. ZM: Conceptualization, Data curation, Writing – original draft, Writing – review & editing.
